# Characterization of community‐wide transmission of SARS‐CoV‐2 in congregate living settings and local public health‐coordinated response during the initial phase of the COVID‐19 pandemic

**DOI:** 10.1111/irv.12819

**Published:** 2020-10-15

**Authors:** Pauline D. Terebuh, Amina J. Egwiekhor, Heidi L. Gullett, Adeola O. Fakolade, Jill E. Miracle, Prakash T. Ganesh, Johnie Rose, Kurt C. Stange, Andrea D. Szabo, Barry Grisez, Kevin Brennan, Suzanne Hrusch, Jackie Napolitano, Ramona Brazile, Terrence Allan

**Affiliations:** ^1^ Cuyahoga County Board of Health Parma OH USA; ^2^ University Hospitals of Cleveland Cleveland OH USA; ^3^ Case Western Reserve University School of Medicine Cleveland OH USA; ^4^ Neighborhood Family Practice Cleveland OH USA

**Keywords:** community transmission, congregate living, COVID‐19, health personnel, local health department, Ohio, pandemic, public health, residential facilities, severe acute respiratory syndrome coronavirus 2

## Abstract

**Background:**

Clusters of COVID‐19 cases amplify the pandemic and are critical targets for intervention, but comprehensive cluster‐level data are not collected systematically by federal or most state public health entities. This analysis characterizes COVID‐19 clusters among vulnerable populations housed in congregate living settings across an entire community and describes early mitigation efforts.

**Methods:**

The Cuyahoga County Board of Health identified and interviewed COVID‐19 cases and exposed contacts, assessing possible connections to congregate living facilities within its jurisdiction from March 7, 2020, to May 15, 2020, during the first phase of the pandemic, while state of Ohio stay‐at‐home orders were in effect. A multi‐disciplinary team‐based response network was mobilized to support active case finding and develop facility‐focused containment strategies.

**Results:**

We identified a cascade of 45 COVID‐19 clusters across community facilities (corrections, nursing, assisted living, intermediate care, extended treatment, shelters, group homes). Attack rates were highest within small facilities (*P* < .01) and large facilities requiring extensive support to implement effective containment measures. For 25 clusters, we identified an index case who frequently (88%) was a healthcare worker. Engagement of clinical, community, and government partners through public health coordination efforts created opportunities to rapidly develop and coordinate effective response strategies to support the facilities facing the dawning impact of the pandemic.

**Conclusions:**

Active cluster investigations can uncover the dynamics of community transmission affecting both residents of congregate settings and their caregivers and help to target efforts toward populations with ongoing challenges in access to detection and control resources.

## 1 INTRODUCTION

The recognition of congregate living settings as high‐risk environments for community transmission and devastating case outcomes was a defining feature of the early phase of the pandemic. Clusters of COVID‐19 cases in congregate living setting emerged as one of the most important determinants of disease spread and the most important opportunity for effective intervention, but comprehensive cluster‐level data are not collected systematically by federal or most state public health entities. In order to effectively characterize and respond to the epidemiology of the COVID‐19 pandemic on the community level, a critical component of case investigations included assessing the potential association with facility clusters. Case counts alone, as aggregated on jurisdictional, state, and federal levels, could not capture the burden of disease or the dynamics of transmission within these clusters or across the community.

In Ohio, despite early stay‐at‐home orders,^1^ and recommendations for limiting access into congregate living facilities,^2^ these settings, as in other states, predominated in early disease amplification.^3^, ^4^ The Cuyahoga County Board of Health (CCBH) jurisdiction has a population of 880,000 and encompasses the entirety of one of Ohio's largest counties excluding the city of Cleveland. An array of congregate living settings exists across the community. CCBH was well‐positioned to partner with these facilities to evaluate factors that contributed to disease burden and to establish effective interventions to contain the spread of COVID‐19 during the early phase of the pandemic. This report describes the community‐wide congregate living‐associated burden of COVID‐19 disease and a comprehensive, team‐based response strategy employed by CCBH during the early phase of the pandemic.

## 2 METHODS

Through long‐standing community partnerships and mandated reporting, CCBH identified and interviewed COVID‐19 cases and exposed contacts and assessed possible connections to congregate living facilities within its jurisdiction from March 7, 2020 to May 15, 2020. Upon identification of a cluster (2 or more cases), administrators at the affected facility were contacted by a CCBH physician who served as the primary point person. The cadre of CCBH physicians was augmented by affiliated preventive medicine faculty and residents as a part of the response surge strategy. Each facility was asked to complete a questionnaire to characterize the facility's size, ownership, and staffing policies and practices, submit a floor plan, and provide a daily line list for both resident and healthcare worker laboratory‐confirmed, probable, and suspected cases under investigation. The lead CCBH physician held regularly scheduled conference calls with facility administrators and a multi‐disciplinary, inter‐professional team consisting of public health staff with participation of community partners, state public health, a regional health system coordinating agency, and, if indicated, local clinical health system representatives.

The response networks were quickly mobilized through the coordination of a dedicated resource manager, activating pathways established through strategic planning during the pre‐pandemic era. These structured team meetings provided situational awareness; assessed screening protocols for both residents and staff; explored testing options and strategies; reviewed contact tracing protocols and findings; explored quarantine, isolation, and cohorting approaches; reviewed environmental hygiene recommendations and strategies for patient transport; and assessed needs for internal and external education and messaging support. The discussions aimed to formulate an effective, facility‐focused outbreak response while cultivating a trusted and supportive partnership.

In response to needs identified during consultation, CCBH and its partners provided supplemental services. Facilities in need of personal protective equipment (PPE) submitted a request form that helped to guide the equitable distribution of limited PPE supplies through the Cuyahoga County Emergency Operations Center. For facilities requiring high consultative support, CCBH physician, sanitarian, and communications staff performed on‐site environmental assessment. In addition, low‐resource facilities unable to coordinate testing for residents, or for healthcare worker staff for whom testing was not readily available through the private sector, CCBH physicians provided a safety net by conducting targeted and mass specimen collection on‐site at facilities or off‐site for individuals for whom the result would impact the outbreak management at the facility. RT‐PCR testing of these specimens for COVID‐19 was performed in most cases by a laboratory at the county hospital with which a community‐focused relationship had been nurtured.

This report describes data collected as part of the local public health emergency response to the COVID‐19 pandemic. Case counts of healthcare workers and residents (detainee or inmate), and information from facility surveys, conference calls, and ongoing consultation were compiled from congregate living facilities with clusters (2 or more cases). Long‐term acute care hospitals, non‐residential hospice, and homeless shelters with which CCBH partnered as part of its community testing strategy, but were located outside the jurisdiction, were excluded from this report.

## 3 RESULTS

We identified 45 facilities with COVID‐19 clusters within congregate living settings in the CCBH jurisdiction from March 7 to May 15 (Table 1) and placed an additional 14 facilities with a single case on a watch list. These clusters accounted for 377 confirmed and 17 probable cases among residents (including inmates and detainees) of whom 79 died, and 167 confirmed and 37 probable cases among healthcare workers (Table 1).

**Table 1:** Characteristics of COVID‐19 clusters in congregate living setting—Cuyahoga County Board of Health Jurisdiction, Cuyahoga County, Ohio, March 7‐May 15, 2020

Cases^a^	No. (% of total cases N = 598)
Resident Cases	
Confirmed	377 (63)
Probable	17 (3)
Healthcare Worker Cases	
Confirmed	167 (28)
Probable	37 (6)
Deaths	
Residents	79 (13)
Healthcare workers	0

Clusters	No. (% of total clusters N = 45)
Facility type	
Nursing home	23 (51)
Assisted living	5 (11)
Corrections	3 (7)
Group home	9 (20)
Treatment Facility	3 (7)
Shelter	1 (2)
Intermediate care	1(2)
Facility Capacity	
*≤*16	11 (24)
17‐59	5 (11)
≥60	29 (64)
Facility Ownership	
County or state	4 (9)
Corporate	16 (64)
Small private enterprise	11 (24)
Non‐profit organization	8 (18)
Unknown	6 (13)
Staffing policies	
Union workforce	4 (9)
Utilized agency/PRN staffing	25 (56)
Maintained dedicated staff	10 (22)
Multiple sites^b^	2 (4)
Unknown	4 (9)
Testing Strategy	
*Symptomatic testing*	
Residents only	20 (44)
Residents and staff	6 (13)
*Mass testing*	
Residents only	5 (11)
Residents and staff	10 (22)
*Unknown*	4 (9)
Index Case^c^	
Resident	3 (7)
Healthcare Worker	22 (49)
Uncertain	20 (44)
Resident Attack Rate^d^	
<10%	17 (38)
10%‐29%	11 (24)
>=30%	7 (16)
No residents affected	9 (20)
Unknown	1 (2)
PPE Availability^e^	
Requested	35 (78)
Not requested	8 (18)
Unknown	2 (4)

^a^ Laboratory‐confirmed case is a person with a COVID‐19 detected by RT‐PCR at any laboratory, and a probable case is a person with compatible symptoms and close contact with a laboratory‐confirmed case.

^b^ Staff deployed at multiple sites within a single organization.

^c^ Case whose date of onset occurs at least one incubation period before other cases in the associated cluster.

^d^ Attack rate among facility residents = number of resident cases/total number of residents in the facility.

^e^ Requested = facility that completed a request form and submitted to the county emergency operations center.

**Figure 1:**
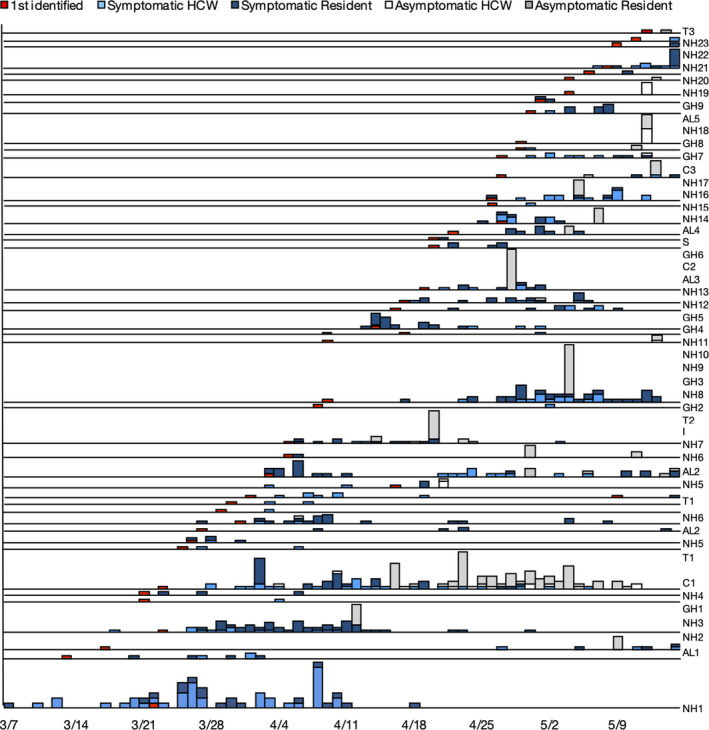
Number of probable and confirmed COVID‐19 cases^†^, by onset date^‡^ among 45 congregate living clusters (N = 598)^§^—Cuyahoga County Board of Health Jurisdiction, Ohio, March 7‐May 15, 2020. Abbreviations: NH = nursing home/skilled nursing facility; AL = assisted living; C = corrections (juvenile and adult); GH = group home; T = treatment facility (psychiatric or drug); S = shelter; I = intermediate care (adults with developmental and intellectual disabilities). ^†^cases were laboratory‐confirmed and probable. ^‡^cases were plotted with their respective facility cluster epidemic curve by date of onset for symptomatic cases and by date of specimen collection for asymptomatic cases. ^§^date of onset was missing for 38 cases

Clusters occurred in correctional facilities, nursing homes, assisted living facilities, intermediate care facilities for developmentally disabled, extended treatment facilities, long‐term shelters, and group homes (Figure 1). The facility size (resident capacity) varied greatly (range 3‐352); however, the sum of individuals associated with a facility also included healthcare workers who, in most facilities, outnumbered the residents. The cluster size ranged (2‐145), with the largest clusters occurring in facilities with the greatest capacity, that is, government correction facilities and large corporate‐owned nursing homes, but many (11) occurred in small settings, typically owned by non‐profit organizations or small private enterprises.

Attack rates were highest within small facilities (chi‐square *P* < .01) in comparison with medium and large facilities. The three large facilities with high attack rates required extensive support (frequent phone consultation, specimen collection and testing assistance, PPE supplementation, on‐site environmental assessment) in order to implement effective containment measures. The workforce within a facility was unionized (4), dedicated (10), but was most often (25) a mix of full‐time, part‐time, and as‐needed (PRN) staff who were sometimes provided by a staffing agency. Test strategies differed for the residents vs. healthcare workers, targeting symptomatic individuals only or in combination with mass testing.

**Figure 2:**
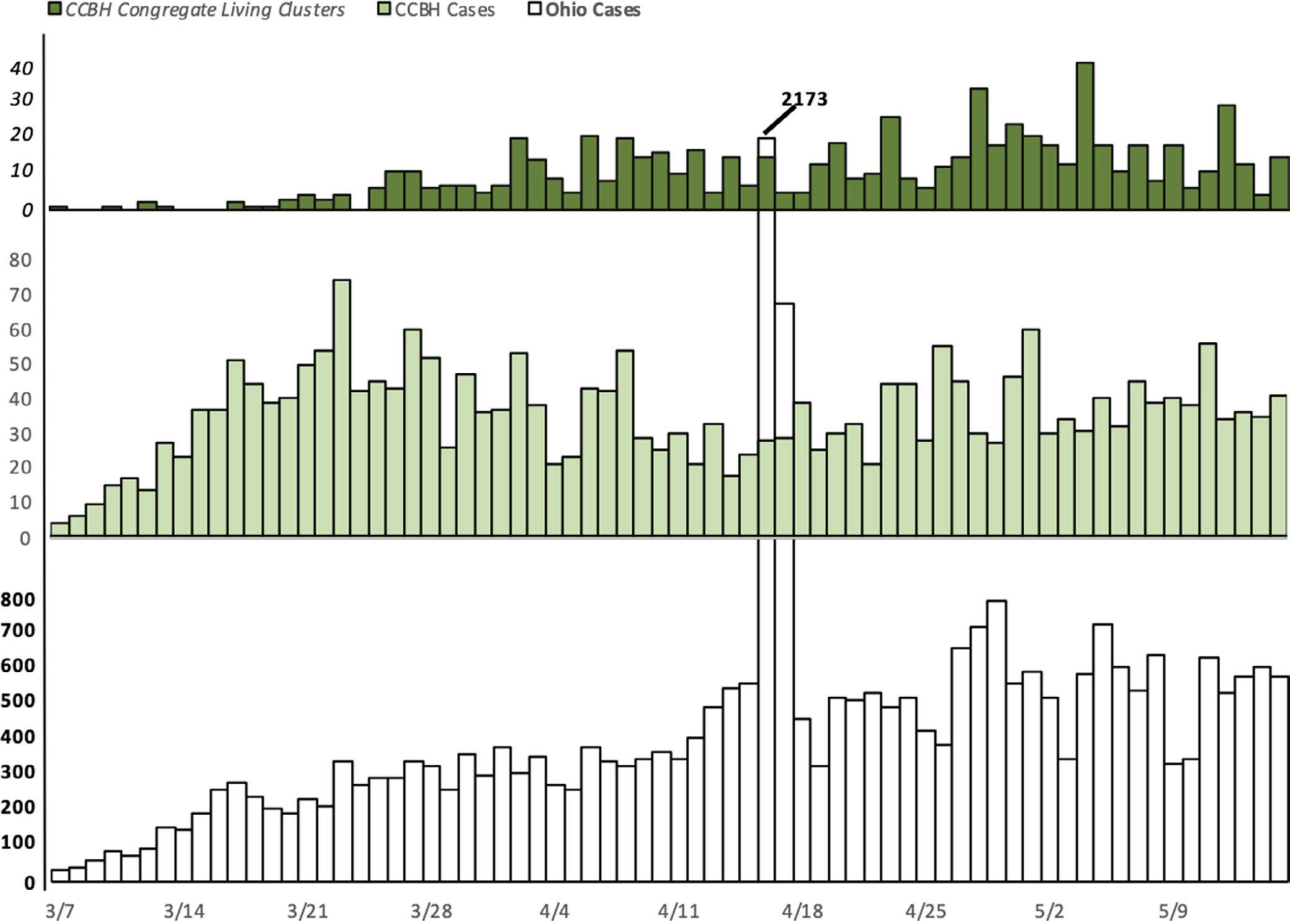
Number of probable and confirmed COVID‐19 cases associated with congregate living facilities in the Cuyahoga County Board of Health jurisdiction, among residents of the Cuyahoga County Board of Health jurisdiction, and among residents of the state of Ohio*—March 7‐May 15, 2020. Abbreviations: CCBH = Cuyahoga County Board of Health. † Cases counts for Ohio residents shown at 1/5 scale

Investigation of the first case identified within the cluster led, in some instances, to the identification of cases with earlier onset dates. When possible (25 clusters), we designated the index case (a single case at least one incubation period before subsequent cases) who was frequently (88%) a healthcare worker. Three healthcare workers were associated with more than one cluster (2 with 2 separate clusters, 1 with 3 separate clusters). Figure 2 depicts a composite epidemic curve of all COVID‐19 cases that constituted the congregate living facility‐associated clusters in the CCBH jurisdiction and the epidemic curves of all CCBH and Ohio cases for context.

## 4 DISCUSSION

We identified a large number of congregate living‐associated clusters of cases early in the COVID‐19 pandemic that drove the high rates of community transmission and that provided vital opportunities for early intervention. Communities have a diverse ecosystem of congregate living settings that house vulnerable populations at risk for COVID‐19.^2^, ^3^, ^4^, ^5^ CCBH attempted to characterize the burden of disease in these settings across the entire community during the first phase of the pandemic when state of Ohio stay‐at‐home orders were in effect. While the number of healthcare worker and resident or detainee cases (598) within these clusters was substantial in comparison with the overall case counts for the CCBH jurisdiction (2325) as of May 15 (Figure 2), many healthcare workers live outside the jurisdiction and those cases do not contribute to the CCBH case counts, making occupational transmission difficult to reflect by those standard metrics. Therefore, facility‐level data are critical for understanding community‐level transmission and dynamics. The rigorous, active case finding methods employed by CCBH allowed these transmission webs to be uncovered.

Staff often were thought to have been the vector for introducing COVID‐19 into the facility, but it frequently was difficult to identify the index case. The variable incubation period and the possibility of asymptomatic undetected infections and multiple simultaneous introductions into facilities contributed to the challenge. Active investigation and case finding in facilities allowed for the identification of instances when the source of infection was not clear. This, in turn, provided CCBH the opportunity to characterize the spectrum of COVID‐19 illness that included pre‐symptomatic and asymptomatic cases, and unusual presenting symptoms that were not well understood during the early days of the pandemic.^5^, ^6^ Once the first case within a facility was identified and laboratory‐confirmed, overlooked illnesses with earlier onset dates, usually with very mild or atypical symptoms, were sometimes identified among staff and/or residents. Their identification underscored the challenge for facilities in identifying cases by symptom screening alone.^2^, ^6^

Active engagement of the congregate living facility leadership with the multi‐disciplinary team created learning opportunities for all team members. It provided an active ongoing forum to educate the facility leadership on emerging COVID‐19 epidemiology and guidelines in order to speed adoption of effective interventions, identify the evolving needs within the facilities, prioritize scarce resources, engage hospital partners in the community, expose representatives of state agencies to frontline challenges, and develop and refine new public health workflows. Above all, it reinforced the strong partnership with public health at a time of crisis.

Common challenges were identified not only within facilities, but also across facilities. The effects of the pandemic highlighted the ongoing hardship of low‐paid workers in congregate living situations who often must work multiple jobs (at multiple sites) to survive financially, thus serving as potential vectors between sites.^3^ Several instances were identified in which these healthcare workers were associated with clusters at multiple facilities. Quarantining of exposed staff created challenges for both the facility and the affected healthcare workers. Policies related to sick leave and personal time off had implications for the willingness to quarantine, especially among the PRN staff who were unlikely to be afforded these benefits.

Many of the larger nursing home outbreaks occurred earlier in the pandemic, suggesting that detection and containment strategies had become more robust over time. For facilities with large outbreaks, mass or targeted testing, identifiable on the epidemic curves in Figure 1 by single day detection of a large number of asymptomatic infections, often heralded the end of the cluster within 1‐2 incubation periods. As the pandemic progressed, these larger facilities were usually able to identify resources for testing their residents, but often lacked a mechanism for testing staff, resulting in an ad hoc approach to case ascertainment. Some healthcare workers were able to obtain testing through their primary care providers, but many were turned away for not meeting the priority testing criteria of the time. CCBH offered testing to these individuals as part of the community testing strategy because identifying their status had potential impact for the management of clusters to which they may have been connected. Additionally, group homes and smaller congregations often had the highest attack rates and the most limited response options, typically lacking a corporate parent. Smaller facilities were less likely to be able to tap a deep staffing roster, have a laboratory contract, or have the ability to cohort and isolate residents, making CCBH their primary resource.

With the lifting of the stay‐at‐home orders, cases have begun to emerge in other sectors of the community. The expanded availability of testing, the growing scientific understanding of the SARS‐CoV‐2 virus and its transmission, the identification of at‐risk populations and effective personal protective equipment (PPE), the stabilization of PPE supply chains, and the experience accrued by all stakeholders made it possible to phase out the intensive intervention stage. Partnerships with local hospital systems for COVID‐19 testing and response had been established for larger facilities, allowing public health to consolidate focus on underserved communities, group homes, and other community clusters while still maintaining enhanced reporting from larger congregate living facilities.

COVID‐19 is a newly emergent disease whose roadmap for response is under construction. Documenting the cascade of clusters among both residents and employees of facilities across the entire community during the initial phase of the pandemic was enabled by a robust local public health force whose capacity encompasses affiliated preventive medicine faculty and residents. Epidemiologic tools created through this process were promptly and iteratively shared with other local health jurisdictions in the state, often constituting the leading edge for local response approaches. While the CCBH strategy of active engagement with congregate living facilities significantly augmented case finding, many other factors influenced disease detection and control, including the evolution in scientific understanding of the spectrum and transmissibility of COVID‐19, considerable day‐to‐day heterogeneity in the availability of testing, access to PPE, and adherence over time to community mitigation orders.^7^ Furthermore, the challenge of identifying new symptoms among elderly with comorbidities and the occurrence of asymptomatic disease likely resulted in cases that went undetected, except for those among individuals who were part of mass testing populations.^6^ ^.^ The precise proportion of disease related to these clusters within the CCBH jurisdiction was difficult to ascertain because some healthcare worker cases identified are counted by other jurisdictions and, likewise, some CCBH cases are likely related to clusters outside our jurisdiction.

CCBH congregate living cluster investigations provided a foundation for early pandemic response. After fostering capacity within congregate living facilities and their relationships with clinical entities, CCBH was able to recalibrate the intensity of support. Local public health, through its ongoing engagement with both the residents and the social and occupational networks within its jurisdiction, will be the most nimble entity in identifying interconnections and in responding to the needs of the community, especially its most vulnerable citizens, as more clusters emerge in the community outside of congregate living. These relationships help to sharpen the focus of potent community resources while plugging the inevitable gaps in the evolving systems that are being created to respond to the pandemic.

## CONFLICTS OF INTEREST

All authors report no conflicts of interest related to this article.

## AUTHOR CONTRIBUTION

**Pauline Terebuh:** Conceptualization (lead); Data curation (lead); Formal analysis (lead); Investigation (equal); Methodology (equal); Visualization (lead); Writing‐original draft (lead). **Amina Egwiekhor:** Formal analysis (supporting); Investigation (equal); Methodology (supporting); Writing‐original draft (supporting). **Heidi Gullett:** Conceptualization (supporting); Methodology (lead); Project administration (equal); Supervision (lead); Writing‐review & editing (supporting). **Adeola Fakolade:** Investigation (equal); Writing‐review & editing (supporting). **Jill Miracle:** Investigation (equal); Writing‐review & editing (supporting). **Prakash Ganesh:** Investigation (equal); Writing‐review & editing (supporting). **Johnie Rose:** Investigation (equal); Writing‐review & editing (supporting). **Kurt Stange:** Conceptualization (supporting); Writing‐review & editing (lead). **Andrea Szabo:** Investigation (equal); Writing‐review & editing (supporting). **Barry Grisez:** Methodology (supporting); Writing‐review & editing (supporting). **Kevin Brennan:** Methodology (supporting); Writing‐review & editing (supporting). **Suzanne Hrusch:** Methodology (supporting); Writing‐review & editing (supporting). **Jackie Napolitano:** Investigation (supporting); Methodology (supporting); Writing‐review & editing (supporting). **Ramona Brazile:** Supervision (supporting); Writing‐review & editing (supporting). **Terrence Allan:** Conceptualization (supporting); Methodology (equal); Project administration (equal); Supervision (supporting); Writing‐review & editing (supporting).

### PEER REVIEW

The peer review history for this article is available at https://publons.com/publon/10.1111/irv.12819.
